# Novel Coatings to Minimize Bacterial Adhesion and Promote Osteoblast Activity for Titanium Implants

**DOI:** 10.3390/jfb11020042

**Published:** 2020-06-16

**Authors:** Samira E. A. Camargo, Tanaya Roy, Patrick H. Carey IV, Chaker Fares, Fan Ren, Arthur E. Clark, Josephine F. Esquivel-Upshaw

**Affiliations:** 1 Department of Restorative Dental Sciences, Division of Prosthodontics, University of Florida College of Dentistry, Gainesville, FL 32610, USA; scamargo@dental.ufl.edu (S.E.A.C.); BCLARK@dental.ufl.edu (A.E.C.); 2 Department of Materials Science Engineering, Herbert Wertheim College of Engineering, University of Florida, Gainesville, FL 32611, USA; t.roy@ufl.edu; 3 Department of Chemical Engineering, Herbert Wertheim College of Engineering, University of Florida, Gainesville, FL 32611, USA; careyph@ufl.edu (P.H.C.IV); c.fares@ufl.edu (C.F.); fren@che.ufl.edu (F.R.)

**Keywords:** antimicrobial, implant, coating, cell adhesion, SiC, TiN

## Abstract

Titanium nitride (TiN) and silicon carbide (SiC) adhesion properties to biofilm and the proliferation of human osteoblasts were studied. Quaternized titanium nitride (QTiN) was produced by converting the surface nitrogen on TiN to a positive charge through a quaternization process to further improve the antibacterial efficiency. The SiC required a nitridation within the plasma chamber of the surface layer before quaternization could be carried out to produce quaternized SiC (QSiC). The antimicrobial activity was evaluated on the reference strains of *Porphyromonas gingivalis* for 4 h by fluorescence microscopy using a live/dead viability kit. All the coatings exhibited a lower biofilm coverage compared to the uncoated samples (Ti—85.2%; TiN—24.22%; QTiN—11.4%; SiC—9.1%; QSiC—9.74%). Scanning Electron Microscope (SEM) images confirmed the reduction in *P. gingivalis* bacteria on the SiC and TiN-coated groups. After 24 h of osteoblast cultivation on the samples, the cell adhesion was observed on all the coated and uncoated groups. Fluorescence images demonstrated that the osteoblast cells adhered and proliferated on the surfaces. TiN and SiC coatings can inhibit the attachment of *Porphyromonas gingivalis* and promote osteoblast adhesion on the titanium used for implants. These coatings may possess the ability to prevent the development of peri-implantitis and stimulate osteointegration.

## 1. Introduction

Dental implant treatment is one of the primary choices for the replacement of missing teeth instead of fixed and removable prostheses. However, dental implants are susceptible to peri-implantitis, which causes the destructive inflammation of soft and hard tissues and, when associated with microbial challenge, leads to dental implant failure [1,2,3,4,5].

Biofilms are responsible for plaque formation, leading to dental caries, periodontal disease, enamel demineralization, and peri-implantitis [1]. Bacterial adhesion to a substrate is a multifactorial process that involves the surface properties inherent to both the bacteria and the biomaterial [6,7].

*Porphyromonas gingivalis* (P. gingivalis) is a principal pathogen of human periodontitis [4,8]. *P. gingivalis* also appears in high quantities during biofilm formation on titanium implants and has demonstrated a typical bacterial profile in peri-implantitis. [5,8,9].

The implant bacterial colonization may arise directly on the implantation site or after bone integration into the oral cavity. Bacterial colonization is influenced by many elements, including the oral environment; bacterial characteristics; and material surface properties such as chemical composition, hydrophilicity, topography, and surface energy [4,10,11,12].

A method used to improve osseointegration and generate a better mechanical solidity of Ti-based implants is to coat their surfaces with biocompatible thin layers [12,13]. These coatings can be classified according to their type of action, such as bactericidal, surface adhesion prevention, antimicrobial-eluting, osseointegration increase, or an association of these actions [12,13,14].

Different coatings—including copper, zinc, fluoride, silver, chlorhexidine, and antibiotics—have been used to give antibacterial properties to the dental implant surface [14,15]. However, few studies are available on implant surface treatments that would avoid bacterial proliferation, colonization, and adhesion [4,11,15], as well as stimulate fast osseointegration and improve bone attachment.

An ideal implant/implant coating should possess both antibacterial and osseointegration properties. The objective of this study was to evaluate the influence of titanium coatings [Titanium Nitride (TiN), Quarternized Titanium Nitride (QTiN), Silicon Carbide (SiC), Quarterrnized Silicon Carbide (QSiC)] on monomicrobial biofilm adhesion as well as determine the viability of human osteoblasts in contact with this coated titanium.

## 2. Materials and Methods

### 2.1. Titanium Sample Preparation

One hundred and twenty high-purity titanium (0.9999) disks (11 mm × 2 mm) were cleaned using the following procedure: (1) acetone cleaning in an ultrasonic bath, (2) isopropyl alcohol rinse followed by a compressed nitrogen drying step, and (3) ozone treatment to remove the surface carbon contamination. Hence, all the implants were polished to the same grit size of 600 to ensure a similar surface roughness across the implants.

### 2.2. Coating Process

After the titanium disks were cleaned, groups of 24 disks were coated with either TiN, QTiN, SiC, or QSiC, or left uncoated as a reference.

The TiN deposition was performed with a Kurt J. Lesker CMS 18 (Jefferson Hills, PA, USA) sputter system. A total of 50 nm of TiN was deposited by rf-magnetron sputtering with an alloyed TiN target at room temperature in a pure Ar ambient. The platen for holding the samples was biased at 30–40 V to improve the uniformity and stoichiometry of the deposited TiN.

QTiN substrates were prepared by converting nitrogen atoms on the TiN surface into quaternary nitrogen by submerging the TiN substrates in an acetonitrile (25 mL) and allyl bromide (100 µL) solution mixed for 1 h to quaternize the surface. After quaternization, isopropanol and deionized water (Fisher Scientific, Pittsburgh, PA, USA) rinses were used to remove any excess solvent and reagent.

For the SiC coating, depositions were performed within a plasma-enhanced chemical vapor deposition system (PECVD, PlasmaTherm 790, Saint Petersburg, FL, USA). The corresponding thicknesses of the deposited SiC coating was 200 nm. Methane (CH_4_) and Silane (SiH_4_) were the precursors used for the silicon carbide deposition. The deposition temperature for the SiC was 300 °C and the deposition rate was 170 Å/min.

The conversion of the surface of the SiC to SiN was performed by treatment with ammonia plasma for 30 min at 300 °C and 400 W. The quaternization of the surface nitrogen was performed using the same procedure as for the QTiN substrates to give a QSiC surface.

### 2.3. Experimental Design

The five groups (n = 120) in this study were: (1) uncoated titanium disks as the control group; (2) TiN-coated titanium disks (TiN-disks); (3) quaternized TiN-coated titanium disks (QTiN-disks); (4) SiC-coated titanium disks (SiC-disks); and (5) quaternized SiC-coated titanium disks (QSiC-disks).

### 2.4. Salivary Pellicle Formation

Twenty-four titanium disks from each group (coated with TiN, QTiN, SiC, or QSiC, and uncoated) were sterilized in an autoclave (121 °C, 60 min), and distributed on a sterile 24-well plate. Each disk was incubated in 1 mL of artificial saliva (Fusayama/Mayer, Stabilized, USA) at room temperature for 2 h. Then, they were washed with sterile distilled water to eliminate the excess saliva and unbound proteins and the disks were air-dried overnight.

### 2.5. Bacterial Growth

To study the antimicrobial properties of the coated and uncoated disks, monomicrobial reference strains (ATCC—American Type Culture Collection) of *Porphyromonas gingivalis* (ATCC 33277) were utilized. *Porphyromonas gingivalis* was chosen for this study because this bacteria is one of the principal human pathogens found in peri-implantitis. The strains were grown onto agar plates with a brain heart infusion broth (BHI-Himedia) for 24 h at 37 °C. After the growth, each microbial suspension was centrifuged at 4700 rpm for 10 min (MPW-350) to separate the supernatant and microbial suspension. The centrifuge process was performed twice to minimize the quantity of debris. After separation, the microbial suspension was adjusted to 10^7^ CFU/mL and was ready to be placed onto the coated and uncoated titanium disks. After dispensing the bacteria solution on the titanium disks, a glass coverslip was placed on top of the bacteria to evenly disperse the solution across the full area of the sample.

### 2.6. Fluorescence Assay for Bacteria

After the incubation period, the bacteria adhering to the titanium samples were stained with SYTO^®^ 9 dye (Live/Dead BacLight Bacterial Viability Kit, ThermoFisher Scientific, Waltham, MA, USA). A Zeiss Imager A2 microscope captured fluorescence images of the live bacteria. The images were then analyzed using the ImageJ software package. Five random areas of each sample were used to calculate to the average bacteria coverage on each filter specimen (n = 8).

### 2.7. Scanning Electron Microscopy

The coated and uncoated titanium groups were examined using an SEM microscope (FEI Nova 430, JEOL, Hillsboro, OR, USA) to determine the bacterial adhesion. After the coated and uncoated disks were incubated, the culture medium was removed, and the monomicrobial biofilm adhering to the samples was placed in a fixation solution of 3% glutaraldehyde, 0.1 mol/L sodium cacodylate, and 0.1 mol/L sucrose for 45 min. The samples were soaked for 10 min in a buffer solution (0.1 mol/L sodium cacodylate and 0.1 mol/L sucrose). The sample surfaces and biofilm were treated in serial ethanol dehydrations for 10 min each, dehydrated in hexamethyldisilazane (HDMS), and stored in a desiccator until SEM imaging. Then, the samples were sputter-coated with a palladium-gold alloy (Polaron SC 7620 Sputter Coater, Quorum Technologies, Lewes, UK) to reduce charging effects during the analysis.

### 2.8. Biocompatibility Testing

Titanium disks from each group were sterilized in an autoclave (121 °C, 60 min) and each one was distributed on a sterile 24-well plate. Human Osteoblasts (Nhost, Lonza, Walkersville, MD, USA) were cultivated in polystyrene vented tissue-culture flasks (surface area 175 cm^2^) at 37 °C in 5% CO_2_. Dulbecco’s Modified Eagle Medium (DMEM), supplemented with 10% fetal bovine serum and 1% penicillin/streptomycin, was used. The cells of passage 3 to 10 were used for subsequent experiments. A quantity of 20,000 cells/well were seeded on the titanium samples in 24-well plates at 37 °C in a 5% CO_2_ for 24 h. Then, the media was removed, and adhered cells were stained with Rhodamine Phalloidin (Invitrogen, ThermoFisher Scientific, Carlsbad, CA, USA) and DAPI (4′,6-diamidino-2-phenylindole, ThermoFisher Scientific, Waltham, MA, USA) according to the manufacturer’s instructions. The cells adhering to the samples were placed in a fixation solution (3.7% formaldehyde in PBS) for 10 min at room temperature (RT). Then, the samples were placed in a solution of 0.1% Triton X-100 in PBS for 5 min. The samples were stained with Rhodamine Phalloidin for 20 min, following by DAPI solution for 5 min at RT. The solution was removed and rinsed with PBS, and the samples were stored in PBS away from light and wrapped in aluminum foil at 4 °C in the fridge. Fluorescence images of the cells were recorded on a fluorescence microscope (Zeiss Imager-A2, Dublin, CA, USA) and analyzed by ImageJ software. The average of the cell percentages was performed over five random areas on each filter sample (n = 8).

The quantitative data were reported as the means ± standard deviations. The statistical differences were analyzed using a one-way ANOVA and Tukey’s test (Graph Prism 6.0, GraphPad Software Inc., San Diego, CA, USA). For all analyses, the statistical significance was pre-set at α = 0.05.

## 3. Results

### 3.1. Bacterial Growth

**Figure 1:**
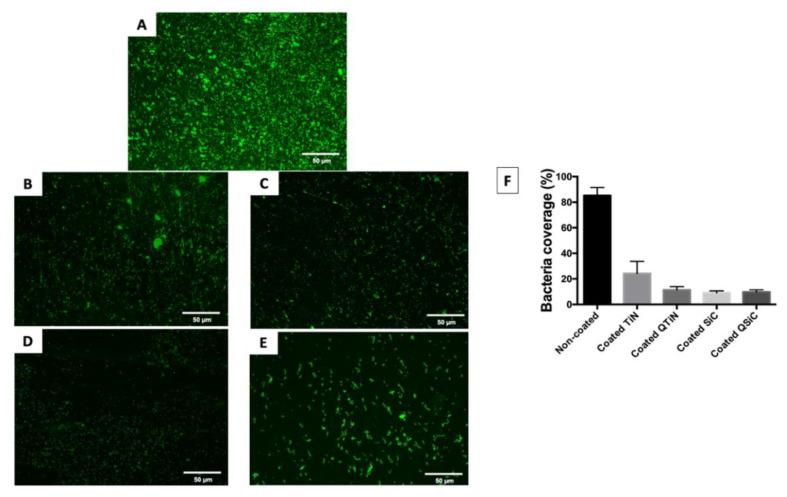
Live fluorescence images of *P. gingivalis* cultured for 4 h on the uncoated and coated titanium disks. The cultures were stained with SYTO^®^ 9 to dye the living bacteria green. (**A**) Uncoated; (**B**) TiN-coated; (**C**) QTiN-coated; (**D**) SiC-coated; (**E**) QSiC-coated. The *P. gingivalis* coverage was calculated from at least 6 fields/disk at 10× *g* magnification using ImageJ software (**F**). Scale bar = 50 µm.

After 4 h, the amount of monomicrobial biofilm (Porphyromonas gingivalis) was less on all the coated (TiN, QTiN, SiC, and QSiC) than the uncoated samples. The samples that were coated with SiC and QSiC showed a biofilm coverage of 9.1% and 9.74%, respectively, whereas the uncoated samples showed a significantly higher biofilm coverage of 85.2% (*p* < 0.0001) (Figure 1).

The biofilm coverage was higher in TiN (24.22%) compared to the QTiN (11.4%), SiC (9.1%), and QSiC (9.74%) coatings, which presented similar quantities of biofilm (*p* = 0.1321). These data suggest that the TiN and SiC-based coatings minimized the adhesion of a particular human pathogen associated with peri-implantitis. After 4 h of culture, the fluorescence images in Figure 1 demonstrated a higher *P. gingivalis* adhesion on the uncoated group than on the coated groups.

**Figure 2:**
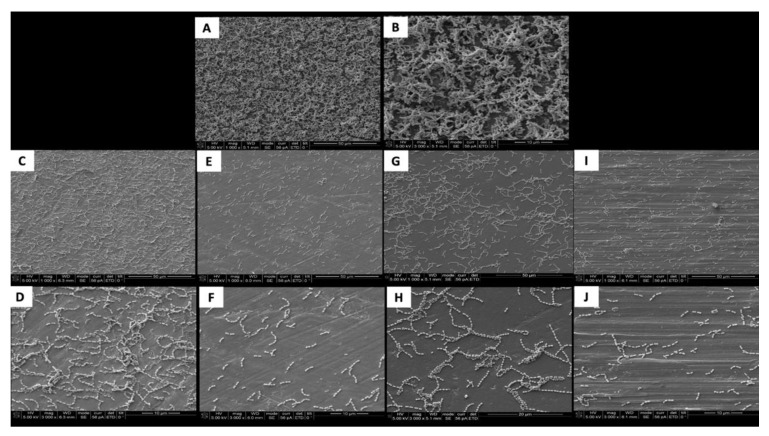
SEM adhesion of *P. gingivalis* after 24 h of cultivation on the uncoated and coated titanium disks. (**A**,**B**) Uncoated; (**C**,**D**) TiN-coated; (**E**,**F**) QTiN-coated; (**G**,**H**) SiC-coated; (**I**,**J**) QSiC-coated.

The SEM images show the results from the live assays, demonstrating a decrease in the number of bacteria adhesions on the coated groups for *P. gingivalis* after 24 h of culture (Figure 2).

### 3.2. Biocompatibility Testing

**Figure 3:**
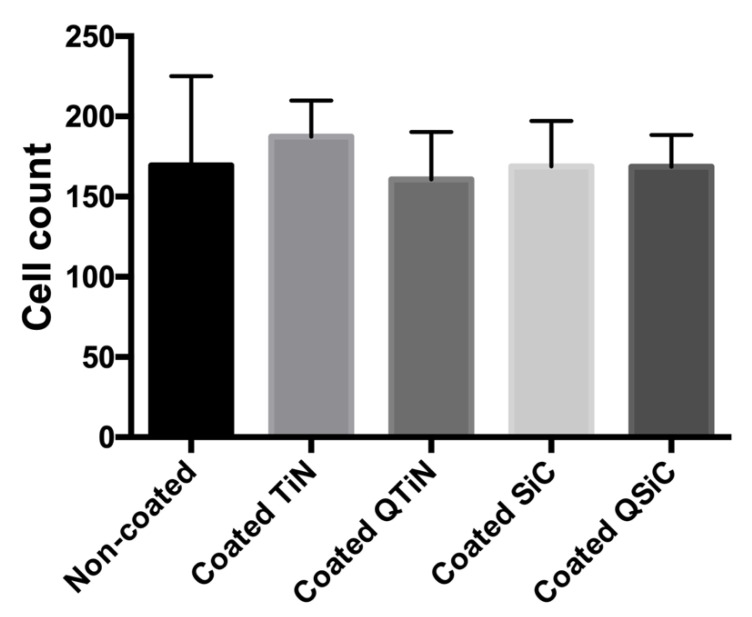
Cell proliferation after 24 h of culture on the uncoated and coated (TiN, QTiN, SiC, QSiC) titanium disks. The cells were quantified from at least 6 fields/disc at 10× *g* magnification using ImageJ software.

To determine whether the coatings impaired the cell proliferation, a fluorescence assay was performed. After 24 h of human osteoblast (Nhost, Lonza, USA) cultivation, the samples that were coated with TiN, QTiN, SiC, or QSiC showed a cell proliferation similar to that of the uncoated group (*p* = 0.1589) (Figure 3).

**Figure 4:**
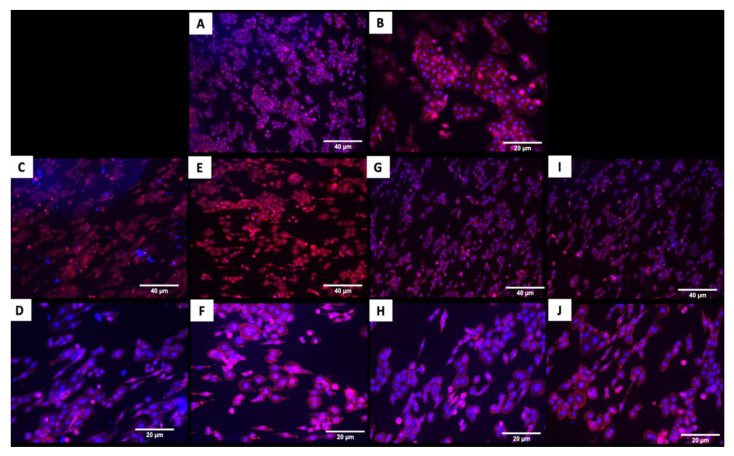
Adhesion of human osteoblasts (Nhost) on uncoated and coated surfaces after 24 h by fluorescence evaluation. (**A**,**B**) Uncoated; (**C**,**D**) TiN-coated; (**E**,**F**) QTiN-coated; (**G**,**H**) SiC-coated; (**I**,**J**) QSiC-coated. Scale bar = 40 µm and 20 µm.

The fluorescence images in Figure 4 demonstrated that cells adhered and covered the surface of the disk after 24 h in culture. Additionally, the cell morphology was similar for cells cultured on the coated and uncoated samples, where the Nhosts appear to be oval-shaped and flattened on the surface.

## 4. Discussion

The physicochemical properties of the material surface, such as roughness, hydrophobicity, and surface-free energy, may interfere with the bacteria attachment. The literature reported that mainly roughness and hydrophobicity stimulate bacterial adhesion [10,15,16,17,18,19,20,21,22,23]. The oral environment possesses irregular surfaces where more bacteria are retained than in smooth areas, as the bacteria are protected from salivary fluid flow, tongue, and muscle action. Thus, smooth surfaces are less likely to be associated with bacterial adhesion [15,24,25,26]. Our previous studies showed that QTiN and SiC coatings were more hydrophobic than TiN and uncoated surfaces, presenting lower bacteria adhesion [27,28]. Additionally, the surfaces of the SiC coatings were smoother than the uncoated samples by the AFM and SEM analyses [28].

In vitro studies have been performed on bacterial adhesion to titanium implants [10,16,24,25,27,29,30,31]. *P. gingivalis* was chosen for this study because these bacteria are a common human pathogen found in peri-implantitis. The adhesion of *P. gingivalis* was verified to be lower on the surface of titanium coated with TiN, QTiN, SiC, and QSiC. Additionally, the percentage of biofilm coverage was eight times less for the titanium coated with QSiC (9.74%) compared with the uncoated group (85.2%) after 4 h. Here, we can demonstrate a positive effect of coatings on titanium surfaces preventing early bacterial attachment, which is desirable for better healing of the implant site.

The reduction in bacterial adhesion may be explained by the ability of quaternary nitrogen atoms to rupture the cell wall, causing the leakage of the cell substances and eventual apoptosis of the bacteria. Our preliminary study exhibited the potential anti-microbial action of quaternized TiN compared with Ti and TiN at decreasing colony forming units and bacteria coverage [27]. Additionally, lower bacterial adhesion and biofilm formation on titanium nitride and zirconium nitride implant surfaces was shown compared with the control samples [30]. Fang et al. [31] verified that a novel hexapeptide-based coating for titanium can inhibit the *P. gingivalis* adhesion due to the carbon-fluorine bond of the fluorinated aromatic ring.

Titanium has shown excellent results for cell adhesion and proliferation, which is essential to favor greater cell anchorage, facilitating osteointegration [13,32,33]. The present study demonstrated that coated and uncoated titanium samples showed a good adhesion of human osteoblasts on their surfaces, and the cell coverage area was similar in both the coated and uncoated samples. However, the coated surfaces showed a more elongated morphology for actin staining (Figure 4), which is one of the proteins responsible for important cell processes like cell growth and contractility [32]. This could contribute to an even better long-term cell adhesion and growth in the coated implants.

The biocompatibility or the cell viability can be evaluated by the direct interaction between the material surface and the cells [32,33], and live/dead studies exhibit good cell biocompatibility. The quantification of cell counts by ImageJ as fields of view (Figure 3) verified that the cell viability of the implants was not less than but similar to the cell viability of the uncoated implants. Therefore, we infer that the implants’ coatings were not detrimental to the osteoblasts and did not lead to cell death. Thus, TiN or SiC-based coatings can promote a suitable substrate for extracellular spaces, as well as the production of proteins, which are believed to have a significant influence on the cell adhesion and proliferation of osteoblasts.

Additionally, the osteoblast adhesion and proliferation on the titanium-coated or uncoated surfaces found in the present study is the first stage to promoting bone formation and consequently the osseointegration of the implant into the bone.

The principal findings of this study are as follows: (a) titanium nitride and silicon carbide-based coatings significantly reduced the bacterial adhesion and prevented dental biofilm formation, and (b) nitride or silicon carbide-based coatings promoted osteoblast adhesion and proliferation. Therefore, these coatings exhibited both osseointegration and antimicrobial properties.

## 5. Conclusions

The nitride or silicon carbide-based coatings increased antibacterial activity and minimized bacterial adhesion capacity without compromising the biocompatibility of the titanium surfaces. These findings could have a clinical impact, as the proposed coatings can minimize the colonization of implant surfaces by *P. gingivalis* and other pathogens. This could potentially decrease the risk of peri-implant disease. This study also demonstrated that the coating did not interfere with osteoblast cell adhesion and proliferation, which are important in promoting bone formation in the implant site.
